# Climate-Aware and IoT-Enabled Selection of the Most Suitable Stone Fruit Tree Variety

**DOI:** 10.3390/s21113867

**Published:** 2021-06-03

**Authors:** Juan A. López-Morales, Juan A. Martínez, Manuel Caro, Manuel Erena, Antonio F. Skarmeta

**Affiliations:** 1 Department of Information and Communications Engineering, Computer Science Faculty, University of Murcia, 30100 Murcia, Spain; skarmeta@um.es; 2 Odin Solutions S.L., Polígono Industrial Oeste C/ Perú, 5, 3º, Oficina 12, 30820 Alcantarilla, Spain; jamartinez@odins.es; 3 Institute of Agricultural and Food Research and Development of Murcia-IMIDA, Mayor Street, 30150 La Alberca, Spain; manuel.caro@carm.es (M.C.); manuel.erena@carm.es (M.E.)

**Keywords:** cold units, interoperability, IoT platform, smart agriculture, stone fruits, weather

## Abstract

The application of new technologies such as the Internet of Things offers the opportunity to improve current agricultural development, facilitate daily tasks, and turn farms into efficient and sustainable production systems. The use of these new technologies enables the digital transformation process demanded by the sector and provides agricultural collectives with more optimized analysis and prediction tools. Due to climate change, one of the farm industry’s problems is the advance or decay in the cycle of stone fruit trees. The objective is to recommend whether a specific area meets the minimum climatic requirements for planting certain stone fruit trees based on climatic data and bioclimatic indicators. The methodology used implements a large amount of meteorological data to generate information on specific climatic conditions and interactions on crops. In this work, a pilot study has been carried out in the Region of Murcia using an IoT platform. We simulate scenarios for the development of stone fruit varieties better adapted to the environment. Based on the standard, open interfaces, and protocols, the platform integrates heterogeneous information sources and interoperability with other third-party solutions to exchange and exploit such information.

## 1. Introduction

Bioclimatology is the science that studies the effect of meteorological variables on living beings and aims to analyze the relationship between the biosphere and the atmosphere, as well as its influence on the troposphere. To establish these relationships, indexes and algorithms are developed to explain the evolution and history of life on the planet. Agroclimatology is a discipline that deals with the adaptation and development of plant species in different climates so that techniques can be established to take better advantage of the plant–atmosphere relationship.

Changes in average temperatures and precipitation, together with an increase in short- and long-term extreme events, are already affecting crop yields in Europe. To adapt European agriculture to extreme weather events, it is crucial to provide weather information that is useful for farmers to avoid losses due to these phenomena and choose crop varieties adapted to climate variability because extreme events during the flowering process are dangerous for many of them [1].

Each crop variety needs a specific combination of climatic conditions to reach its potential; their cycles are closely linked to particular events that occur at certain times [2]. When this does not happen, crops react. Increased temperatures cause fruit trees to flower earlier and develop faster, so early varieties with less of a need for chilling hours appear on the market [3].

On the other hand, the appearance of varieties with low winter cold requirements has led to the establishment of these crops in warmer areas, dedicated until a few years ago to the cultivation of vegetables, so that the start of the harvest is considerably earlier. This precocity in warm areas causes the use of varieties with lower cold requirements than the usual ones used in traditional growing areas, leading to very high economic losses due to frost. Thus, the choice of conventional varieties in warmer regions, such as the coastal zone, can cause physiological imbalances that lead to poor flowering and consequently to a decrease in the harvest.

Due to the unstable weather or the increase of extreme climatic phenomena, such as frost, agricultural insurance companies’ indemnities to compensate for losses produced in stone fruit tree crops have increased. In Spain, payments made reached 132 million euros during 2020, 15% more than the previous year, with a total affected area of 46,940 hectares [4]. Due to the increase in claims, there is an urgent need to incorporate mechanisms that adapt insurance to the new climatic conditions.

The technological revolution transforms everything, and not even the agricultural sector escapes this situation, which is why connectivity and digitization play a decisive role in the agri-food sector. The digitization of agriculture will allow data to be collected and exchanged at an unprecedented level. Note that the nature of agricultural data is peculiar yet diverse [5]. The collection of this data includes, among others, agronomic, terrestrial, climatic, and even compliance data.

The digital transformation is a challenge, and the continuous technological advances that are currently taking place: Internet of Things (IoT), big data, artificial intelligence, and aerial images, to name a few, are providing the agricultural sector with new tools that are helping to determine the real needs of farms, also improving their efficiency [6]. Specifically, IoT has changed the traditional paradigm of access and management of sensors and actuators due to all productive elements’ connectivity, thus achieving the integration of real information and knowledge in the digital world [7].

These technologies also enable farmers to combine multiple data sets to make more efficient use of natural resources and achieve higher agricultural yields [8]. As a result, it is possible to establish crop alternatives in different locations based on changing agroclimatology to achieve an appropriate balance between farm costs and crop yields. The use of data-driven technologies is advancing rapidly with IoT implementations. These developments will become an essential part of the agricultural sector’s future by improving the efficiency and output of the agri-food sector [9]. Besides, the digitization of the ecosystem through IoT enhances the adoption of new varieties, optimal crop management, and economic benefits to agriculture.

Fruit trees have strong environmental conditions, mainly related to temperature. Therefore, the temperature conditions the tree’s productivity, so using a climatically unsuitable variety in an area conditions the productivity. This work aims to define a system that helps in the decision-making in the varietal choice in an area based on weather conditions, experiences from other agricultural years, field notebooks, and plant sensors. For this purpose, it is necessary to calculate the current conditions that determine the different species’ behavior and varieties of stone fruit trees. The system is a helpful tool for making decisions on the degree of climatic adaptation of varieties to each zone, leading to an increase in tree productivity. It allows anticipating scenarios for early warning on the appropriateness of placing stone fruit trees in areas based on their cold requirements and the risk of frost.

In the following sections, we first describe related work on cold requirements in stone fruit trees and IoT to help determine the best varietal group in an area. Then, we show the datasets used and the methodology followed to give the recommendations. Finally, we detail the results obtained in the Region of Murcia and the system’s conclusions.

### Physiology Models in Stone Fruit Trees

In the annual development cycle of deciduous fruit trees, there is a period in which the plant inhibits the growth, at least visibly, of any meristematic structure as a form of defense against frost. This rest or dormancy period is called latency or dormancy and is composed of two stages—endodormancy and ecodormancy, which develop successively, although they overlap in the intermediate period [10]. To overcome the first stage, the plant needs to be exposed to low temperatures depending on the species and variety. In addiction to temperature, the incident radiation, measured as sunlight duration, influences this resting period exit. In the second stage, the plant needs to accumulate a specific number of hours with warm temperatures that favor this period’s break.

Knowledge of both processes’ evolution is essential to evaluate the date of flowering, especially in self-sterile varieties that require cross-pollination; it is also of particular interest in breeding programs. It is possible to estimate species and varieties’ adaptation from the exit of dormancy, depending on the expected date of flowering and ripening, to different climatic environments.

There are two methods for determining the duration of dormancy: the experimental and the statistical method. In the former, first described by Bennett et al. [11], samples of branches with buds are collected sequentially during the winter and placed in a controlled growth chamber to check the response of the buds. The statistical method is based on the study of historical series of hourly temperature records and phenological observations in the field, such as flowering date, as described by Tabuenca et al. [12].

Algorithms for determining the onset and duration of this stage of deciduous fruit crop physiology are based on hourly mean temperature records. They can be either quantitative or qualitative. The first to be used, quantitative, is the ’Chilling Hours’ model described by Weinberger et al. [13] in which the number of hours with temperatures between zero and seven degrees Celsius is counted. This method is still used today because of its simplicity, but it does not conform to experimentally determined values.

To overcome these discrepancies, models were developed based on hourly mean temperature records and quantitative nature. The “Utha” method, developed by Richardson et al. [14], establishes positive, negative, and null values for the latency output as a temperature range function. Cold accumulation occurs between 2.5 and 12.5 ${}^{\circ }$C, outside of which collection is null or negative. This method is very well adapted to physiology, but only in cold climates; in warm climates such as the Mediterranean, it does not have a high ratio [15].

More recent is the “Dynamic” model [16], which considers that cold accumulates in the buds of deciduous fruit trees irreversibly, provided that a critical concentration has been reached at an intermediate stage depending on the intensity and duration of the temperature. Furthermore, it considers the importance of alternating temperatures within a cooling cycle. The nullification of the cooling effect by high temperatures depends on the process’s level and duration when alternating with low temperatures.

## 2. Related Work

The consequences of the new climatic scenarios on fruit production, particularly on stone fruit trees, are transcendental in all temperate climate producing areas, especially in relatively warm regions such as the Mediterranean area, affecting adaptation and phenology. Furthermore, these changes mean that fruit growers face weather-related risks due to the high probability of adverse agro-climatic phenomena in Europe affecting crop phenology [17]. Nowadays, there are already tools such as the one proposed by Dheebakaran et al. [18] that provide weather-based agricultural advice for multiple crops and different crop growth stages.

Climate changes generate significant interest in developing specific tools and models adjusted for each type of crop. The study by Buerkert et al. [19] determines that the reduction of winter cold affects crop development, advising the use of more suitable ones; for this purpose, it uses a combination of temperature values and stochastic simulations for past and future temperature scenarios. For fruit species in temperate zones, phenological models have been proposed and contrasted for their predictive capacity in different climatic scenarios, such as the one offered by Chuine and Régnière [20]. Serrano-Notivoli et al. [21] have developed a package based on the R language that provides bioclimatic indices to assess potential climate impacts on crops. In the same line, the work of Miranda et al. [22], also based on R, simplifies the evaluation of climate adaptation and the identification of potential risks for fruit species.

Increased temperatures have two direct consequences that affect fruit production: delayed cold winter accumulation in the autumn–winter period and reduced cold winter accumulation. Tomczyk et al. [23] show that the number of frost days in spring tends to increase from western to eastern Europe despite a warming trend. Although the lack of cold hours can delay flowering, the delays can be compensated by a more rapid accumulation of warmth for the plants in spring, as shown separately by Luedeling and Eike [24]. This combination of cold and heat modeling has been mainly applied to different fruit trees species, as explained by Fadon et al. [25].

Solutions are now available for estimating cold requirements in stone fruit trees. As dormancy is highly dependent on temperature, it can potentially impact phenological evolution and production in the sector. The study by Ding et al. [26] explores methods of modeling the cause–effect relationship in frost forecasting. Watteyne et al. [27] define an IoT-based system using a peach frost prediction engine based on historical data. We can also find works, such as Elijah et al. [28], that describe the benefits and challenges of IoT in agriculture to monitor variables that affect crops such as air, temperature, humidity, and soil moisture level.

The use of techniques based on Artificial Intelligence, together with IoT, allows improving the prediction of variables, as is the case of the system proposed by Ponce and Gutiérrez [29]. The system uses supervised learning to predict the temperature; the result is compared with a web service, obtaining an error of approximately 2.1 °C; the model used obtains excellent results but to determine the cooling needs of crops, the error should be lower. In a similar vein, the work proposed by Gutiérrez and Ponce [30] propose a supervised learning method called artificial hydrocarbon networks (AHN) to predict the temperature at a remote location together with the intelligent detection and identification of possible sensor failures; the behavior of the proposed model should be verified with a network of agro-meteorological stations.

Neural networks, too, have been used to predict low temperatures based on time series using various techniques, such as the Autoregressive Integrated Moving Average, used by Castañeda et al. [31]. The system uses a neural network and a fuzzy expert system, the former to optimally predict the temperature inside the greenhouses and the latter to control the activation of a water pump.

Other works focus on developing a decision system based on fuzzy models for frost prediction to inform and alert, such as the one proposed by Cadenas et al. [32]. This work cites a regression technique based on k-nearest neighbors that supports data sets with approximate values.

Spatial data help predict variables such as yield or optimal harvest date, so it is essential to integrate and link them with the rest of the data generated by farms. Bordogna et al. [33] proposes an architecture for managing a spatial data infrastructure to create, collect, and analyze heterogeneous geospatial value sets from multiple sources and time series using web services. Furthermore, the work of Jiang et al. [34] demonstrates that there are various possibilities to integrate statistical modeling techniques and Spatio-temporal data for area-specific crop management. Quinta-Nova et al. [35] propose to identify new areas that can exploit for fruit tree production. The site suitability analysis was carried out using Geographic Information Systems (GIS) and using the analytical hierarchy process as a multicriteria decision analysis technique. Gonzalez et al. [36] present a dashboard used to create yield and fruit quality maps, facilitate farmers’ decision-making, and optimize water, fertilizer, and pesticide use.

## 3. Use Case, Data, And Infrastructure

Every day, increasingly more steps are being taken towards smart agriculture, enabling better land use planning, sustainably transforming agricultural practices, improving risk management for climate change adaptation, and using climate-smart information.

Information becomes the essential ingredient of any technological innovation and a key element for the agricultural sector. The first thing to do is transform “raw” data into processed information to improve decision-making to display helpful information.

### 3.1. Scope Of Study

The Region of Murcia is a single-province autonomous community located in the southeast of Spain, surrounded by Alicante, Albacete, Granada, Almería, and 220 km from the Mediterranean coast. Between parallels 37${}^{\circ }$23 and 38${}^{\circ }$45${}^{\prime }$ N and meridians 0${}^{\circ }$39${}^{\prime }$ and 1${}^{\circ }$20${}^{\prime }$ O. Its surface is 11.317 Km${}^{2}$ and represents two percent of the national territory. The cultivated area is around 315,000 Ha.

It is considered as one of the essential areas in the cultivation of stone fruit trees, apricot (*Prunus armeniaca* L.), cherry (*Prunus civium* L.), Japanese plum (*Prunus saliciana* L.), and peach (*Prunus pérsica* L.). Due to the region’s optimum climatic conditions, most of these species have an excellent adaptation to these conditions, despite the scarcity and irregularity of rainfall, limiting their development. The latest available surface and production data for these species in the Region of Murcia, Spain, and the rest of the producing countries correspond to the year 2019, published by the Ministry of Agriculture, Fisheries, and Food [37], and FAO [38]. They confirm that the Region of Murcia, representing only 16.86% of the Spanish agricultural area for stone fruit trees, represents 21.38% of the production with 23,623 MT. Although the total area devoted to these crops only represents 0.45%, it represents 0.93% of world production.

### 3.2. Data Description

**Figure 1:**
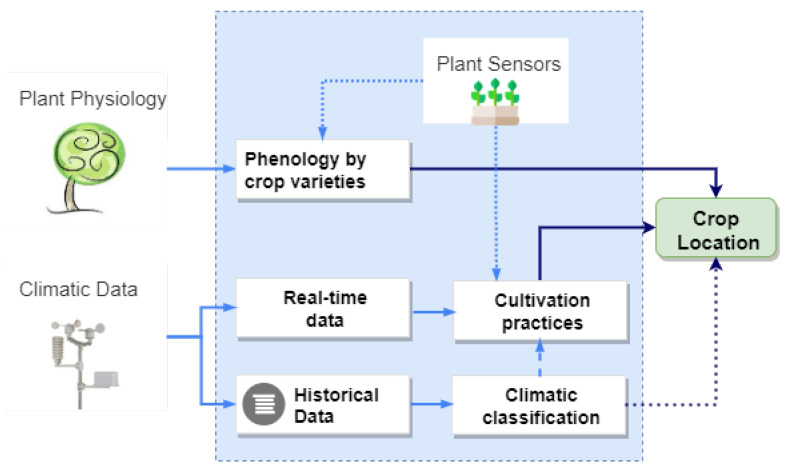
Data sets used and expected results.

In order to know what is the most recommended area for stone fruit tree planting in the given region we need to get some information about the climate of the area and the varieties’ physiology to improve decision-making. In Figure 1, we can see the different sets of data needed and the results that are intended to be obtained from them; in the following sections, we will discuss the treatment carried out with them.

To recommend which varietal group is the most suitable in a given area, we need to obtain two types of data: climatic and physiological. Thanks to the historical climatic data, we can classify the type of climate in the chosen area. We can determine if the crop growth is occurring optimally from the real-time climatic data and the sensors’ data located on the plant if the agricultural practices are correct. From the crop’s physiological data, we obtain the minimum characteristics necessary for a crop to generate the highest yields (kg per hectare) in a given area. The sensors provide us with information from the plant to determine if the crop physiology is developing correctly and if the fertigation is following the patterns indicated for that variety. Thanks to integrating the different data sets, we can recommend the best suitability of an area for a varietal group based on temperature.

#### 3.2.1. Climate Information

First, a climatic classification is obtained during stone fruit tree dormancy (from October to March) in the producing areas. For this purpose, agrometeorological information is collected from the susceptible zones where stone fruit trees have or have had great relevance to make the classification. The stone fruit tree producing areas are located in the Ebro River Bank, Mediterranean Arc, and Southwest Spain.

The Agroclimatic Information System for Irrigation of the Spanish Ministry of Agriculture, Fisheries, and Food [39] is the primary source for obtaining climatic data, but not the only one; data have been obtained from other networks such as

- the Meteorological Service of Catalonia [40] for the province of Lérida,
- the Agroclimatic Information Service of the Government of La Rioja [41] for the province of Logroño, and
- the Agricultural Information System of the Murcia Region (SIAM) [42] for the province of Murcia.

Those stations with a historical series of more than 15 years of hourly records are selected from each of the networks. All the chosen networks store data every half hour, except for the Region of Murcia, which holds data every ten minutes. The stations are installed following the guidelines of the UNE 176101:2010 Standard “Networks of automatic agrometeorological stations. Characteristics, instrumentation, and specific aspects”, which modifies the UNE 500520:2002 [43] Standard, so the temperature is measured at 150 cm and wind at 200 cm. Furthermore, they comply with UNE 500510:2005 “Automatic weather station networks. General aspects and nomenclature”, which governs the minimum guidelines required for the characterization and use of data from automatic meteorological and agrometeorological stations.

In Table 1, we observe the selected municipalities of the production area, the number of fixed stations, and the total number of processed hourly records.

#### 3.2.2. Crop Physiology

Once the climatic data have been collected, it is necessary to check the species’ effects, ecological needs, and physiological response to environmental stress in the different phenological phases. To determine this process, it is necessary to have the information provided by the field notebooks and the support of plant sensors that allow validating the crop’s evolution.

Farm management information systems have evolved from simple farm records to sophisticated and complex production management support systems. Field or farm notebooks help plan, organize, and record all relevant farm data, such as fertilizer applications, fertilization, nutrients supplied to crops, phytosanitary treatments, humidity checks, or anomalous climatic events [44]. These notebooks must be reliable as they represent the farmer’s work manual or guide, bringing him benefits in the short-, medium, and long-term. Typically, they are usually web applications that store the cloud’s information, although they still exist on paper.

Stone fruit trees need to accumulate a specific amount of cold during the winter to overcome the dormancy stage and then experience warm temperatures to flower finally [45]. This process conditions the adaptation of varietal groups in each region, being the major drawback for introducing these crops in warmer latitudes. Therefore, gathering information on each varietal group’s minimum temperature requirements will allow growers to use this information to anticipate the future performance of their farms and the adoption of new varieties in other regions. To obtain this information, we have the information provided by the field notebooks, such as the one shown in Table 2; we can see some of the information that can be obtained from the notebooks, explicitly indicating the cooling requirements for different varietal groups. The accumulated cold base indicator shows the amount of cold that a crop must accumulate to change the phenological phase and start accumulating heat for each variety group.

At the same time, plant sensors are used to monitor crops’ evolution, such as soil moisture sensors, dendrometers, or sensors to obtain the trunk’s hydraulic potential, and thus monitor in real-time the development of crops and the effect of the weather on them. The crop’s vegetative behavior is observed employing remote sensing, relating physical variables obtained from the soil, such as the structural properties of the crops, with the reflectivity or temperature systems of a sensor.

Using these sensors, it is possible to validate and refine the data provided by the networks of stations and field notebooks, and in some cases, to modify the decisions taken.

In our case, we use soil moisture probes to help program irrigation, know the irrigation system’s behavior, and know if there is excessive drainage, which indicates low irrigation efficiency.

Simultaneously, satellite images (Sentinel-2) and the different indices they provide are used to detect plants’ water status for continuous and detailed monitoring of the use made of agricultural land on farms.

### 3.3. Data Analytics

Once the data are stored, several processes are carried out to generate bioclimatic indices and statistics based on the average temperature obtained from each station’s in situ and historical records.

#### 3.3.1. Data Validation Module

This module is in charge of filtering the climate data to verify that the variables are within the previously defined thresholds and detect possible data gaps, marking them inconsistent. Using these filters, it is verified whether an observation is within a predetermined range that can be fixed or dynamic. The fixed ones can be physical (e.g., humidity cannot be higher than 100%) or instrumental (derived from the sensor specifications). The dynamic values are given by the extreme weather records of each area, which is known as meteorological ephemerides.

A data cleaning module is developed to eliminate outliers that may be contained in the raw or real-time sensor measurements regarding data quality assurance. In this sense, outliers are the main problem of climate data quality. To solve data validation, six levels of validation are defined, according to the UNE 500540:2004 standard “Automatic weather station networks: Guidelines for the validation of meteorological records from automatic station networks. Real-time validation”:

1. Validation of the data based on two limits:
  - Rigid (physical and instrumental): The most restrictive of the physical or instrumental limits are applied. Any data outside the established limits will be invalid data.
  - Flexible limits: These limits must be based on the extreme values that the different variables may take in the station’s area. The ideal is to have a set of meteorological ephemerides for each month and season.
2. Validation of the data’s temporal coherence: the change between two or more consecutive observations at ten-minute intervals is compared. If the difference exceeds a preset value, different for each variable, it is considered that the data have not exceeded this level.
3. Validation of the data’s internal consistency (Relationship between sensors): the meteorological relationships that exist between observations made at the same station are taken into account. Values measured at the same time and in the same place cannot be inconsistent with each other. That is, the mean wind speed cannot be greater than the gust.
4. Time series consistency: the mean and standard deviation are calculated for each variable. If the standard deviation is less than an acceptable minimum, all data for that period will be considered suspect. The most considerable difference between any pair of observations of a specific variable in that period should be calculated. If that difference is less than an acceptable minimum, all data will be considered suspect. A visual inspection could discern between feeling the data as valid or invalid.
5. Validation of spatial consistency (contrast of data from each station with data from other stations): to validate spatial consistency, a value is usually estimated for each observation. This task is performed from the same variable’s validated data, recorded at other stations correlated with the variable under analysis. The difference between the measured value and the estimated value is then calculated. If this difference exceeds a certain threshold, it is considered that the analyzed data has not exceeded the level.
6. Visual inspection: to apply this level, it is beneficial to map maximum, minimum, or accumulated values of the different variables and derived parameters. It is also helpful to represent the other variables of temporal evolution at various aggregation levels, especially when trying to determine whether suspicious data is valid or not.

#### 3.3.2. Bioclimatic Indicators Module

The module in charge of generating the bioclimatic indicators incorporates a complex event processing engine and a SQL-based processing language to define and execute the corresponding processes. To address the chilling hour requirements of the varietal groups, it is necessary to estimate the average temperature using the following methodology:

1. The initial date of entry into latency (to start counting cold hours) is obtained for each season and year.
2. Once the starting date is obtained, a review of the dates is performed to determine which data are suspicious, either because of data gaps or inconsistencies. For this purpose, the mean, earliest, latest, median, mode, the difference between the earliest and latest dates, the number of years, and the standard deviation of the set of dates are obtained for each station. Two checks are performed with these data:
  (a) The first check consists of reviewing the stations where the difference between the earliest and latest date is very high, greater than 50 days.
  (b) The second is based on standard deviation values. The highest values occur in warm seasons where there can be a lot of difference between warm or cold autumns and winters, so it is decided to check the seasons in colder areas for any dissonant values. Therefore, data with a deviation more significant than 15 days are checked.
3. Next, we calculate the accumulated weekly cold values based on the Utah and Dynamic or Portions methods of hourly records. For the calculation of the cold requirements in the endodormancy phase, by these two methods, we establish as a starting point the already estimated starting date by studying a series of hourly records of six months until June of the following year, and we develop two steps to establish the end of the count.
  (a) Records are generated by week, season, and year, with the sum of the weekly UF and the portions and their accumulated since the beginning of the count, as well as the number of records with which the values are obtained.
  (b) From the data processed in the first stage, the final date of the count is established, which will correspond in the case of the Richardson method to the first week after which negative or null values are obtained for at least three weeks and in the case of the dynamic model to the last week with positive values.
4. The interannual values are stored with the starting date of the count, by Richardson’s method, in which the statistical variables mentioned in the second section are included.
5. The last step is to estimate the accumulated Cold Units and Portions per week for each season based on the calculations made for each year.

Based on historical data, the probability of extreme events in a year or critical weeks for the development and production of crops is calculated and their evolution with the aim of seeing if they tend to increase significantly with climatic fluctuations. This information will also be added for the characterization of each location. Precisely, three types of extreme events are calculated:

1. Risk of frost, an event is considered to occur when the temperature is −0.5 ${}^{\circ }$C for at least three consecutive hours.
2. Abnormal events, when the temperature is > 21 ${}^{\circ }$C for at least six consecutive hours or when the temperature is > 23 ${}^{\circ }$C for at least three consecutive hours.
3. Anomalous temperatures in the flowering phase, an event is considered to occur when the temperature is > 25 ${}^{\circ }$C for at least three consecutive hours between February and March.

### 3.4. An Open Platform Based on Standards for the Sake of Interoperability

Once the “transformation” of the data into information has taken place, the methodology followed allows us to recommend the location or not of a particular varietal group in an area. However, to offer a more helpful service, it is necessary to use a system that serves as a means to integrate the different data sets, automate the processes generated and visualize the results in a user-friendly environment in a single system.

For this reason, we decided to use an IoT-based platform that promotes the use of standard and open interfaces and protocols that allow the integration of heterogeneous information sources and interoperability with other third-party solutions to exchange and exploit the information. The platform consists of specific modules for integrating IoT devices, historical data exploitation, and a map-based interface. It also allows you to set custom rules to trigger alarms and actions based on the measurements provided by sensors, weather stations, or actuators.

**Figure 2:**
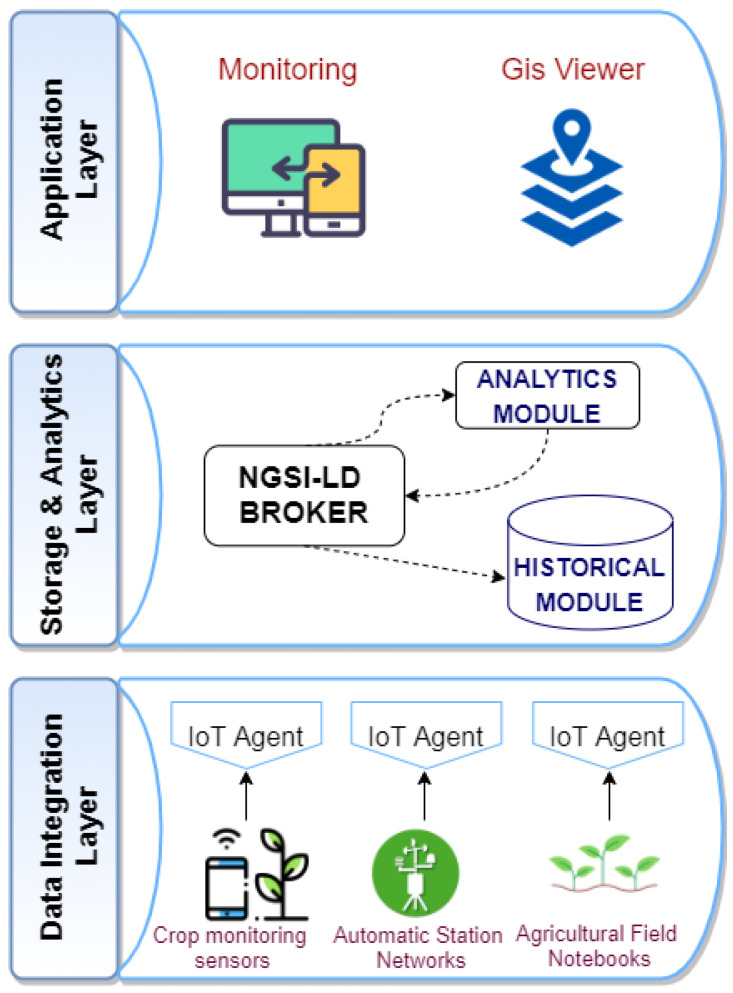
Diagram of the architecture.

The proposed platform’s architecture has a modular layered form, represented in Figure 2: it ranges from the deployment of sensors and monitoring data extraction techniques to the intelligent processing of the data to generate recommendations on crop production in a given area. These layers are based on open and standard initiatives such as the one provided by the FIWARE Community [46] based on the Next Generation Service Interfaces with Linked Data (NGSI-LD) API developed by the European Telecommunications Standards Institute (ETSI). One of the most significant features is its interoperability, so it is possible to integrate the information provided by sensors and other services that use the NGSI-LD interface.

Each of the platform layers is linked to the data processing explained in the Section 3.2:

1. Data Integration Layer is in charge of data collection, connecting third-party devices or databases to the system through Customized Agents.
  Regarding the physical layer, devices can be connected through wired or wireless technologies. For the wired ones, well-known industrial serial connections (typically RS485), direct digital/analog I/O connections, or the use SDI-12 (Serial Digital Interface at 1200 baud) have been integrated. For latter, a wide spectrum of technologies have been considered, ranging from the cellular-based ones such as GPRS or 5G, up to LPWAN technologies considering Sigfox, LoRaWAN, or Narrow Band IoT (NB-IoT).
  The values of the different datasets are integrated into the platform using the FIWARE IoT Agent component that acts as an intermediary between the sensors and the rest of the architecture by providing a uniform API for setting up the configuration and processing of the data. The Agents are in charge of translating known, open, and lightweight protocols (Constrained Application Protocol (CoAP), Message Queuing Telemetry Transport (MQTT), HTTP, and similar), allowing the possibility of integrating new data sources through the use of customs agents. These agents are in charge of transforming the information coming from the devices and sending it to the Broker through NGSI-LD, in both directions to and from the Broker.
2. Storage & Analytic Layer provides storage, analysis, and processing services. Besides, it transforms and prepares the raw data into NGSI-LD information models, allowing the data to be homogenized and processed by the upper layer.
  The first step is to configure the IoT devices in the *NGSI-LD Broker*; this Information Broker exposes an HTTP REST API based on NGSI-LD for both registration and querying, as well as a subscription/notification approach. The *IoT Agent* must then provide the data to it via an NGSI-LD entity in the Broker. All these changes are stored in a non-relational database based on JavaScript Object Notation (JSON) or JavaScript Object Notation for Linked Data (JSON-LD) objects, because they must provide agile and flexible access to the information. At this point, these data can be transmitted so that consumers can subscribe to this information. The NGSI-LD interface is used to send data updates and receive notifications about device changes. The Context Broker allows us to communicate all data from any entity that manages the platform and performs platform updates.
  Then, the historical module is used to integrate the historical series data extending the Broker functionality. This module uses the same information model, so it is unnecessary to generate any harmonization process of the historical data. Furthermore, it has an ad hoc API to retrieve raw historical data from sensors and simple aggregation functions (sum, average, minimum, or maximum in a certain period) on the data.
3. Application Layer is the interface between the platform and the users of the system. Data analysis procedures and their results can be invoked at this level. It consists of specific modules for IoT device integration, essential data mining, and a map-based interface. Information exchange in both directions (from and to) a third-party platform is also possible thanks to API based on NGSI/NGSI-LD. Thus, this architecture can be applied to provide applications to improve agricultural decision-making such as agro-climatic monitoring, climatic/geographical suitability of varietal groups, and crop monitoring. Potential users of the system include farm managers, agricultural producers, researchers, and, to a lesser extent, agrarian insurance companies. Depending on the user, the platform offers different tailored services to ensure that each user receives the most appropriate information, depending on the data needed. Some of the services provided are as follows:
  - User-friendly reports and graphs of the processed data are provided thanks to a beautiful web-based interface.
  - Allows setting customized rules for triggering alarms and actions based on measurements provided by sensors or weather stations.
  - A recommendation engine provides personalized information based on geographical area, bioclimatic indicators, and varietal group suitability.

More technical specifications on the platform’s behavior can be found in the article published by López-Morales et al. [47].

### 3.5. Equipment

The cost of implementing the system has been reduced due to the Open Source feature of the platform. A virtual machine was created under CentOS 8 environment to carry out the related tasks, with four processors (Intel Xeon Sandy Bridge), 32 GB RAM, and a 128 GB SSD hard disk, along with an additional 500 GB disk. The software used was MongoDB database, QGIS, and Python 3.7 (Anaconda environment) using libraries such as scikit-learn, numpy, pandas, and gdal. Good performance is obtained as we can add more resources by modifying the virtual image used thanks to the proposed architecture’s flexibility.

As for the proposed platform, the implementation level achieved according to technology readiness level (TRL) is considered to be close to TRL 6. This level is achieved because technicians have tested the system’s critical functions in farms or agricultural cooperatives. The system is being evaluated by the fruit-growing team of the Institute of Agricultural and Food Research and Development of Murcia (IMIDA).

## 4. Experiments And Evaluation

This section shows the results obtained after evaluating the IoT platform’s functionalities in the Region of Murcia.

### 4.1. Architecture Instantiation

**Figure 3:**
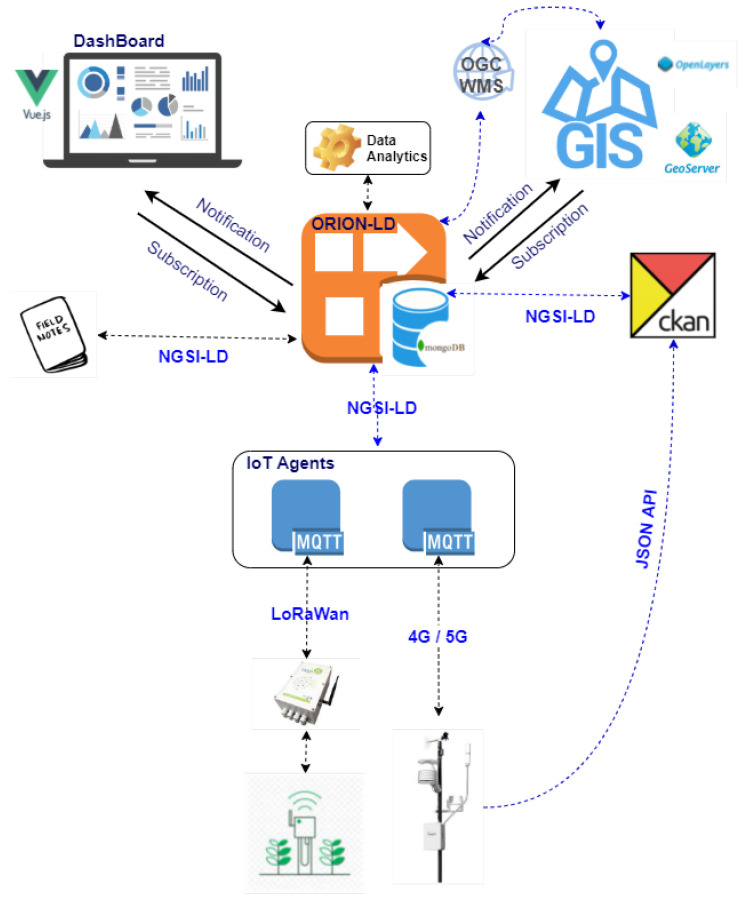
The scheme followed for the implementation of the proposed architecture.

In this section, we are going to detail how the deployment of the IoT-based architecture, Section 3.4, has been carried out to determine the geographical suitability of stone fruit trees based on bioclimatic information. Figure 3 shows the scheme followed to perform such instantiation.

First of all, the sensors to be used in the system must be integrated through the most widely used communication standards NB-IoT, 4G/5G, LoRaWan, or SigFox, which allows data to be transferred securely. Two types of sensors are incorporated through IoT Agents that allow us to retrieve information from the devices, manage the creation of entities in the Context Broker for each device, and act as a producer of attributes on the sensors. The types of sensors incorporated are as follows:

- Soil moisture sensors that take measurements at six depth levels (every 10 cm), Drill & Drop from Sentek [48], responsible for measuring the volumetric water content in the soil. This device is connected via an SDI-12 interface to Odin Solutions’ Mex06 outdoor monitoring datalogger [49]. This datalogger is low power consumption and has different connection modes: RS232, TTL Serial, SDI-12, and, importantly, RF 802.15.4g on which IPv6 over Low power Wireless Personal Area Networks (6LowPAN) operates and is compatible with wireless communication modules (3G, RF, LoRA, or Sigfox).
- Agroclimatic station networks, the vast majority of the climatic stations from which data have been obtained are Campbell Scientific [50] brand. As the stations are equipped with 4G or 5G connectivity (some of them), an IoT Agent over MQTT has been defined to carry the data produced in time to the Context Broker. In addition, the FIWARE CKAN extension has been used to load historical data, which can publish open datasets thanks to the use of RESTful JSON API, together with the ability to provide time series on the context information already generated.

**Figure 4:**
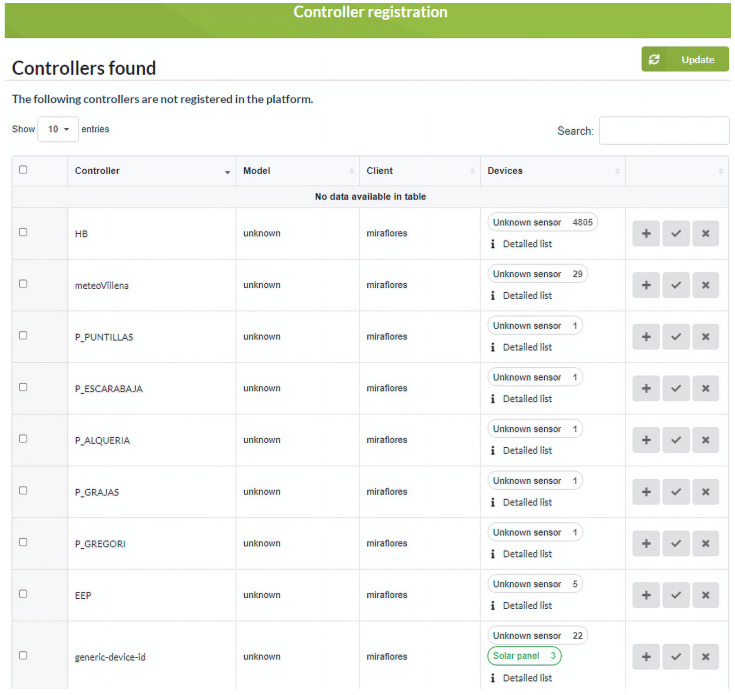
Controller registration module on the platform.

The platform allows managing the registration and de-registration of controllers and the definition of their associated devices. As the communication between the software platform and the controllers is done through the MQTT protocol, it is possible to configure the connection parameters with the MQTT broker from this module. Registration is done using a discovery mechanism that requires the controller to communicate at least once with the platform to be registered. The module can identify for each controller which devices it has: digital inputs, analog inputs, counters, or solenoid valves, from the data received; such identification is possible from the attributes or MQTT identifiers that the controller is sending. Figure 4 shows the registration window where a list appears with all the controllers discovered pending to be registered in the system. This list shows each controller its serial number, controller model, client, and associated device information (inputs and outputs). Each discovered controller has options to edit its configuration, register the controller automatically or delete it. Automatic registration is a quick way to register a controller, creating the controller and associated devices.

After integrating the sensors in the system through the IoT Agents, these are in charge of creating the entities in the Context Broker; in our case, we have used the ORION-LD Broker that supports the NGSI-LD and NGSI-v2 APIs. In addition, CKAN provides new functionalities over ORION-LD as it only has the current state of the entities, so the information is stored in a non-relational database MongoDB. The way to integrate the sensors in the Broker is by using the NGSI-LD interface to send updates on the data and receive notifications about the changes that occur in them. As an example, it is presented how the information provided by the soil moisture sensors is integrated into the platform by the Agent that implements JSON over MQTT. Listing 1 shows the representation of the datalogger that provides connectivity to the sensor:

**Listing 1**. Mex06 datalogger representation.

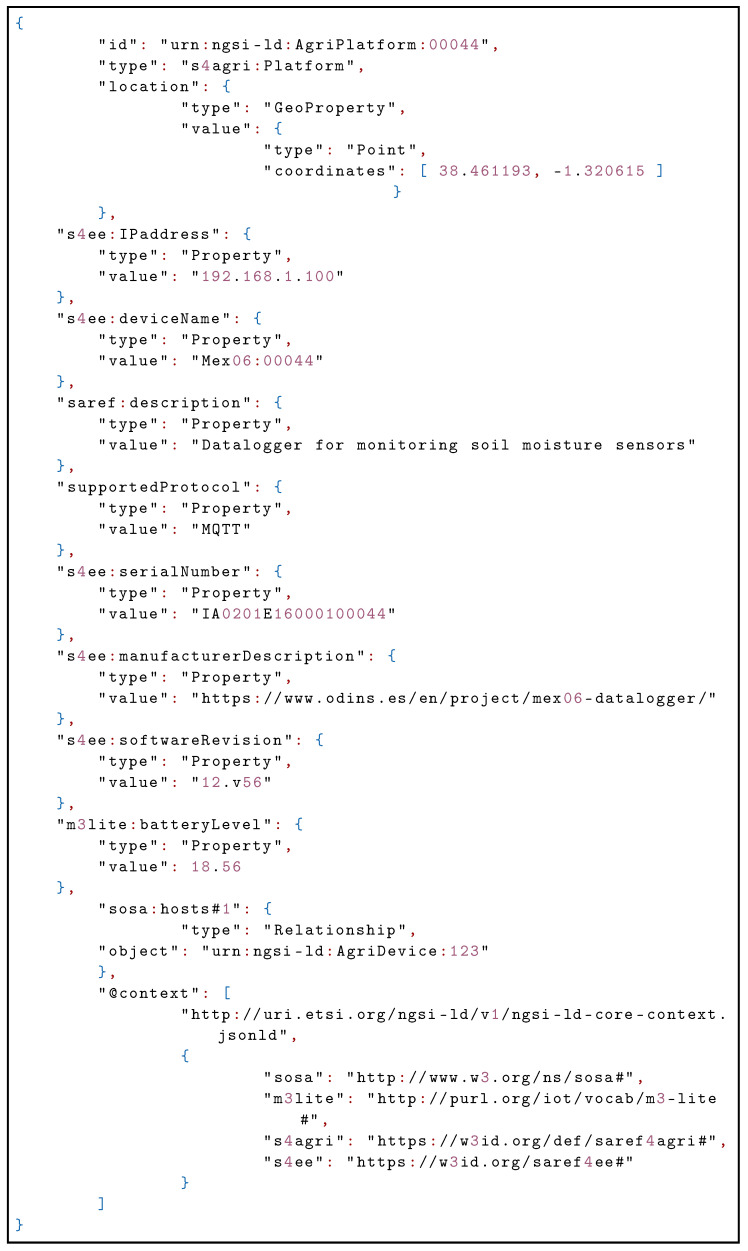



To represent the soil moisture sensor, Listing 2, the first thing to do is to define the essential characteristics of the device:

**Listing 2**. Soil moisture sensor representation.

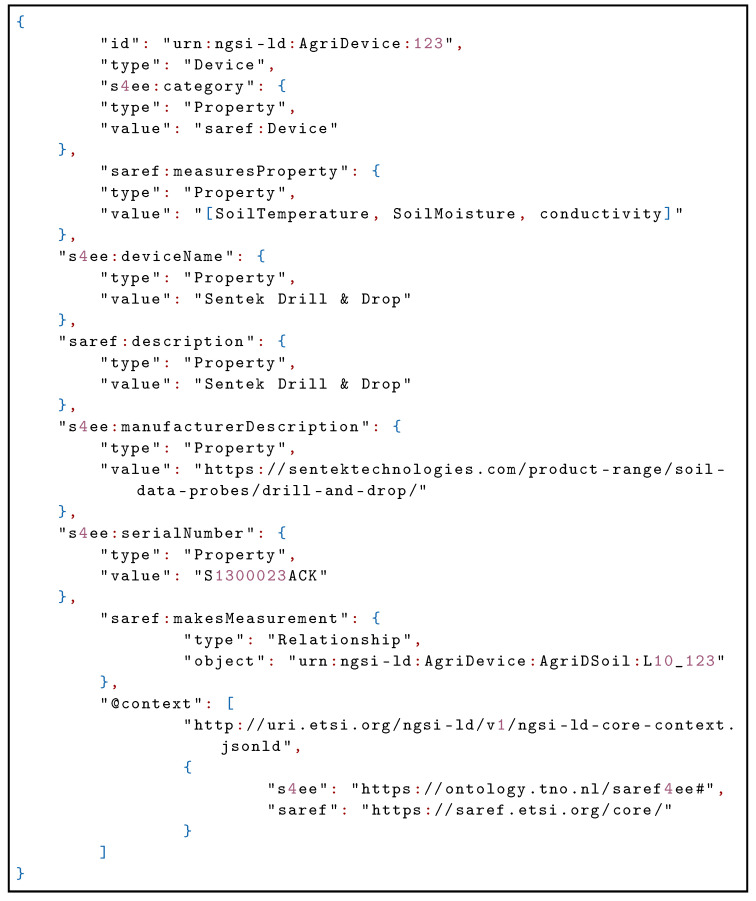



Next, it is shown how the sensor measurements are represented in the different levels, Listing 3. In the Location property, the Z coordinate is added to indicate the depth where the measurement is taken:

**Listing 3.** Measurements taken at each of the set depth levels.

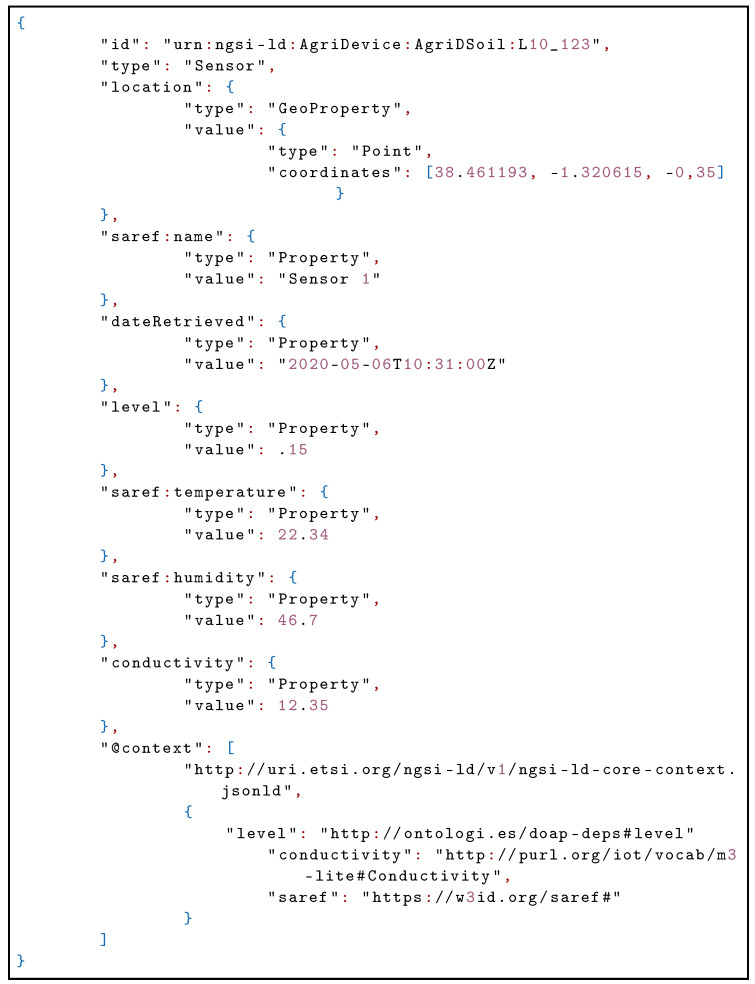



Another dataset used has been the field notebooks used to annotate the biological information of the crops and the optimal water, cold, or heat requirements that the varietal group should accumulate to obtain the best possible production. The field notebooks are directly integrated into the Broker, thanks to an API developed in Python. This API is also responsible for acting as an intermediary between the Broker to generate the necessary analytics, shown in the following sections, and the dashboard that relates all the information generated.

After processing the data, information is generated that determines the geographic suitability of stone fruit trees. These results can be visualized through two mechanisms:

- As a result of the integration of data sources, a *Dashboard* is developed that shows us, in real-time, information related to agroclimatic data, crop evolution, and optimal bioclimatic requirements for each variety. In addition, it allows us to see the events of interest, which have been generated and are reasons for alert in each crop. The module has been developed using the JavaScript framework, Vue.js [51] that allows us to create a backend based on Python, integrating it with front-end web technologies such as HTML, CSS, or JavaScript.
- The integration of a *GIS Viewer* has been realized thanks to both Web Map Service (WMS) and Web Feature Service (WFS) services provided by the OpenLayers technology, so that information can be obtained to characterize the selected region, together with frost risk zoning. The OpenLayers JavaScript library has been used for the GIS viewer and a GeoServer instance connected to the NGSI-LD Broker.

### 4.2. Classification into Climatic Zones

**Figure 5:**
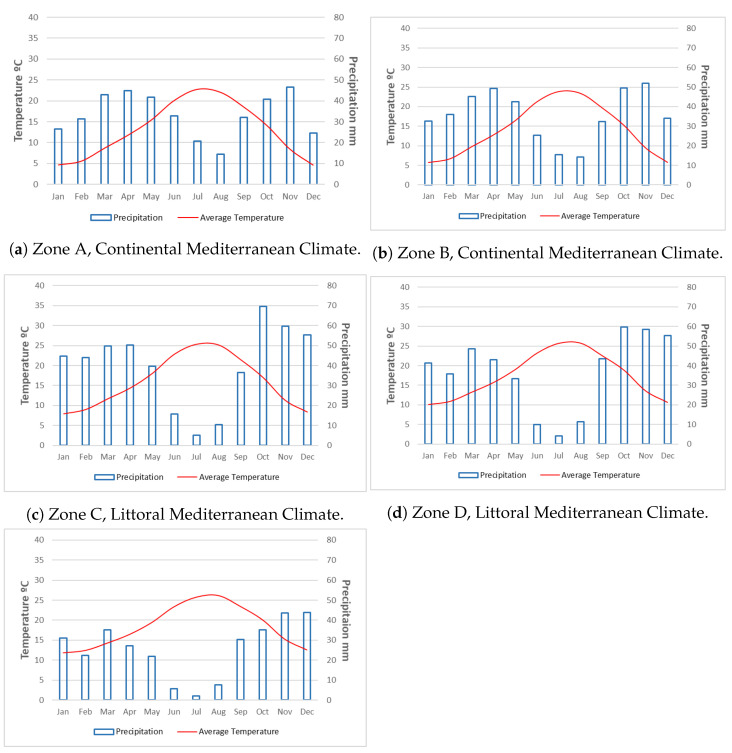
Ombrothermal diagrams for the five determined homoclimatic zones.

One of the first results obtained has been the climatic classification for stone fruit trees in the Region of Murcia to make this classification have been used the results obtained by the Operational Group Stone Fruit. “Innovation Project for the adaptation to climate change in stone fruit” [52], where one of the tasks has been to make a classification of climate zones: extremely warm, warm, intermediate, cold, and extremely cold. To verify these zones’ existence, we generated ombrothermal diagrams for each zone, thanks to the historical data processed, as shown in Figure 5.

The five climates represented in the ombrothermal diagrams mean a temperate climate, according to the Köppen–Geiger classification. The average annual temperatures range from thirteen degrees Celsius (group A) to eighteen degrees Celsius (group E). There are five months in the first two groups, from November to March, with temperatures below ten degrees Celsius. There are only three months from December to February in group C, and in groups D and E, there are no months with average temperatures below this limit.

The annual precipitation regime coincides with that of the Mediterranean climate since the five groups are in the range of three hundred to five hundred millimeters per year. However, the first four groups exceed four hundred millimeters. By months, groups A and B do not have precipitations lower than ten millimeters. Group C has precipitation lower than ten millimeters in July, group D in June and July, and finally, group D has very scarce rains during the three summer months.

As a result of the thermo-pluviometric values, we made a classification, according to Köppen–Geiger, and we consider that the stations have a temperate Mediterranean climate. Those of groups A and B of the Continental Mediterranean type, those of groups C and D of the Littoral Mediterranean type, and those of group E of the Semi-Arid Mediterranean type.

**Figure 6:**
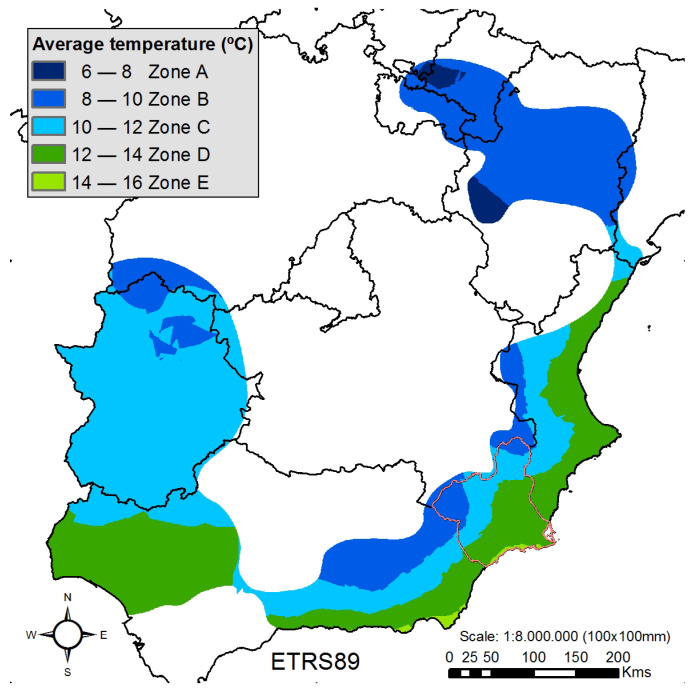
Climatic classification of stone fruit tree growing areas in Spain.

**Figure 7:**
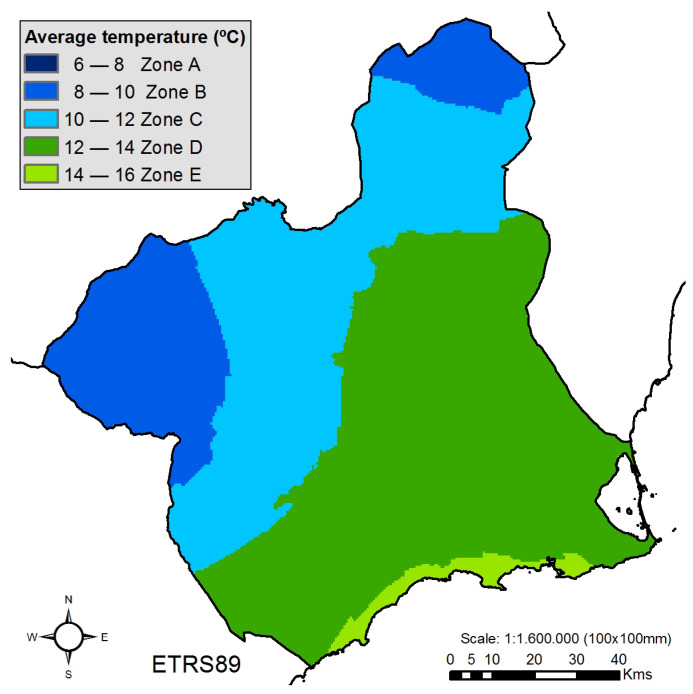
Climatic classification of the stone fruit tree producing areas in the Murcia Region.

Once the climatic classification is done, we generate a map representing the stone fruit tree producing area of Spain (highlighting in red the Region of Murcia), Figure 6, and employing this representation; we make an extrapolation for the Region of Murcia, Figure 7. From where we obtain the classification of the stations of the Region of Murcia according to their location. Table 3, represents the total number of stations for each of the climatic zones and their mean climatic values.

### 4.3. Cold Indicators

**Figure 8:**
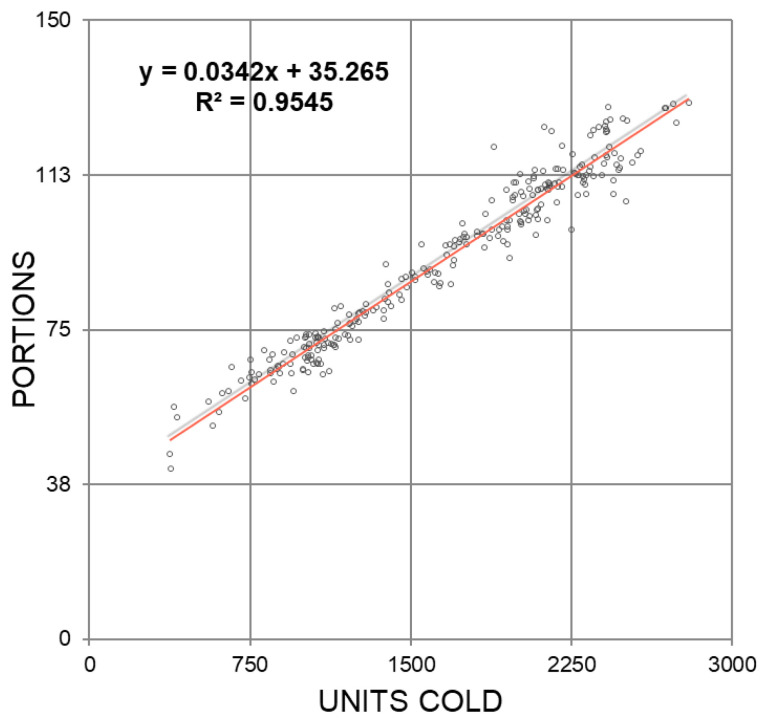
Data sets used and expected results.

Once the Cold Units and Portions have been calculated with the average temperature data, a correlation of both indicators is performed. Figure 8 shows that both methods have a good relationship (R${}^{2}≃$ 0.94) so that neither method distorts the other.

**Figure 9:**
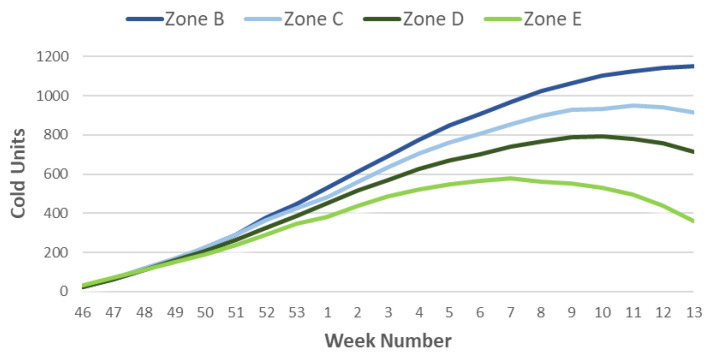
Representation of the Cold Units in the most representative weeks for the climatic zones of the Murcia Region.

**Figure 10:**
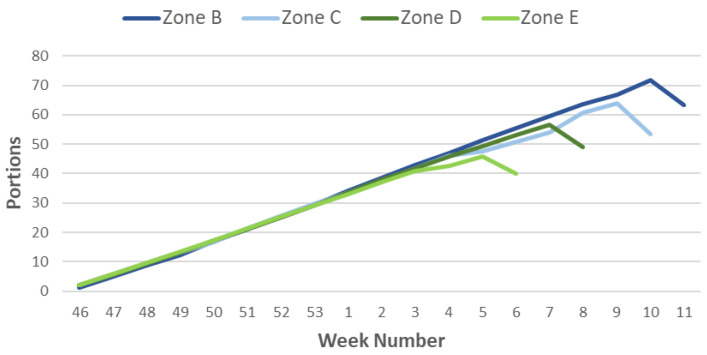
Representation of Portions in the most representative weeks for the climatic zones of the Murcia Region.

Next, the Cold Units indicators (Figure 9) and Portions (Figure 10) are generated for the weeks and zones previously classified (October to March). We observe that the accumulation of the necessary cold in the warm zones occurs earlier than in the rest. Using Richardson’s method, which is better adapted to colder climates, we observe that the period of cold accumulation is shorter than that of the portions, only from week 46 to week 49 or 50, so that depending on the climate, there is a decrease in the weekly accumulation of Cold Units.

Using the Portions method, which is more uniform than the previous one, a similar cold accumulation occurs from week 46 to week 2, when differences begin to be observed depending on the different climates. Thus, zone E, extremely warm, starts the cold accumulation in week three, reaching the end of the count in week five. For the warm weather season, the change in the curve’s slope occurs in week four and continues to accumulate portions until week seven. Zone C corresponds to the intermediate climate, and although the inflection occurs as in the previous one in week four, it continues with the accumulation of cold (portions) until week 9. Finally, in curve B, which corresponds to the cold climate, the inflection point coincides with week eight, much later than in the two previous curves, ending the count in week 10.

The probability and risk of temperatures causing frost damage vary with the sensitivity of the species and varietal group; this knowledge helps farmers decide if, what, and when to plant in a particular location. For this reason, a WMS is created that represents the zoning of the Region of Murcia based on the probability of frost damage to the most representative stone fruit trees. Note that the local conditions of each plot strongly influence the frost damage potential. Furthermore, the geographical assessment of damage potential is a complex problem that needs to integrate a large amount of information. However, it shows a close relationship with altitude, orientation, and coastal influence.

In fruit trees, critical damage temperatures change with the stage of development of the crop. These dates vary from year to year; consequently, determining the probability and risk for fruit plantations is more complicated than for annual crops. The damage that occurs depends on the crop’s sensitivity to freezing at the time of the event, as well as on the time and temperature below the critical damage temperature (Tc). To determine this risk, the theoretical probabilities of occurrence of a temperature as low as Tc are calculated for each sensitive period according to a binomial distribution used by Haan’s method [53]. The Tc (−3 °C) is the one that can cause significant damage between the start and end dates of the most sensitive period of the crop.

For zoning, cadastral polygons are used, which are classified according to the probability of frost occurrence depending on the varieties:

- Group I: Extra-early and Early.
- Group II: Mid-season and Late.
- Group III: Late.

### 4.4. Crop Performance

**Figure 11:**
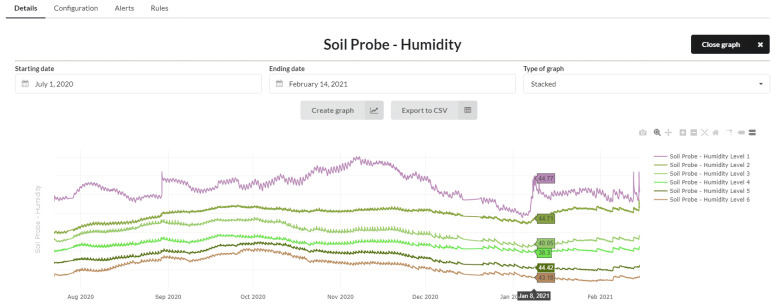
Representation of different soil moisture levels in a peach plot to monitor water stress.

To monitor the volume of water in the soil and check that agricultural practices have been carried out correctly, soil moisture probes are installed at different levels. The sensors or probes are installed in similar areas of the plot to be applied to the whole field. These sensors measure the soil’s water content at a specific moment; the values are expressed in % or mm of water per 100 mm of soil depth (separation between each of the levels). These sensors also allow us to monitor the plant to avoid water stress moments or plant percolation episodes. Figure 11 shows the graph with the evolution of the readings taken by a Sentek sensor after its integration into the platform.

**Figure 12:**
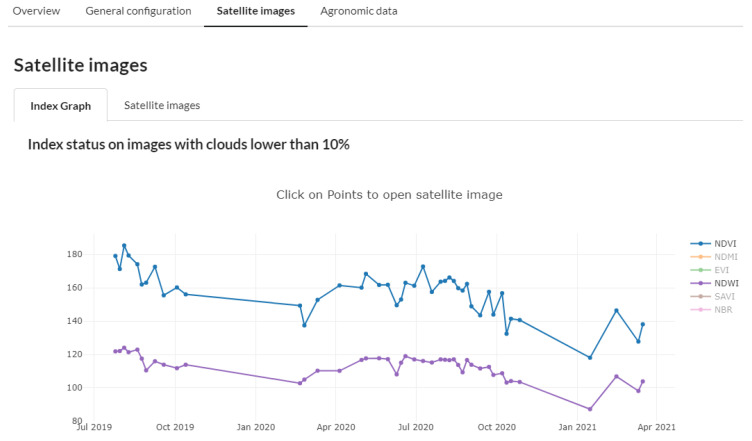
Use of vegetation (NDVI) and water (NDWI) indexes for satellite monitoring of crop condition.

We also use satellite images, in our case Sentinel-2, to extend the monitoring of the vegetative development of the farms and check that the crop is growing according to the data obtained from the field notebooks; for this purpose, we use the Normalized Difference Vegetation Index (NDVI). Similarly, by using the Normalized Difference Water Index (NDWI), we study the spatial distribution of the surface moisture content in the whole study plot, unlike soil moisture sensors that do it in a single point. In Figure 12, together with the NDVI and NDWI values, we can also access the study plot’s satellite image.

### 4.5. Geographic Viewer

Once all the data sets have been processed, the next step is collecting, acquiring, digitizing, georeferencing, and codifying the existing data within a GIS framework. This viewer allows the current data (maps, images, orthophotos, and tables) to be available in an orderly and compatible way to generate derived information to help in decision-making.

**Figure 13:**
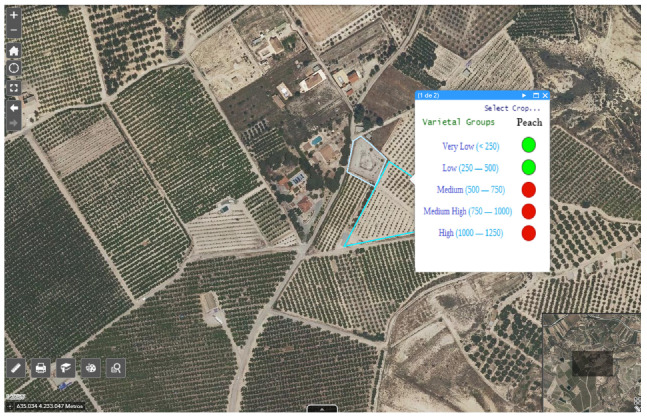
Geographic viewer showing the recommendation for planting varietal groups in an area.

To improve the recommendation in choosing of the best varietal group in a selected area, the advice to implement or not a specific variety in the chosen site has been added in the viewer, based on the cooling needs of each group in the area, see Figure 13.

**Figure 14:**
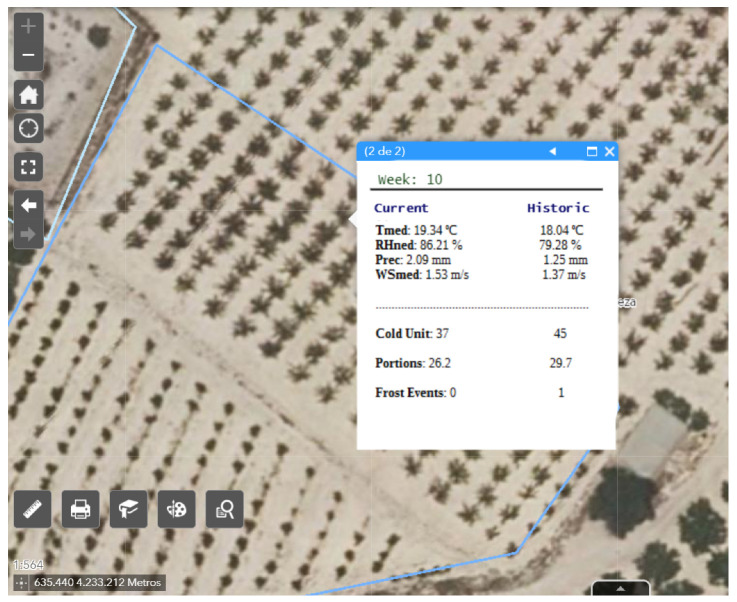
Geographic viewer that shows the evolution of the variables (current and historical) in the area.

Another functionality, more oriented to the farmer, shows throughout the current week the historical and current climatological evolution of the selected area and the data of cold requirements (Portions and Cold Units) as shown in Figure 14. Furthermore, it shows the probability of frost or anomalous events, which improves productivity and avoids economic losses due to not meeting the selected variety’s cold requirements in the chosen area.

## 5. Conclusions and Future Work

In stone fruit trees, the cold requirements and critical damage temperatures change with the stage of development of each varietal group, and the dates of growth stages vary from year to year. Consequently, determining probability and risk for fruit orchards is more complicated than for annual crops.

The work presented here describes an IoT architecture and how to use this architecture to improve decision-making in the agricultural industry and help the sector’s digital transformation. To this end, we propose an interoperable IoT-based platform as an example of integrating various data sources through the use of NGSI-LD and other interoperability standards. The proposed platform gathers a large base of agro-climatic information that is “translated” using easy-to-understand visualizations to the growers’ needs and technicians. This system is presented to the user through an open and interoperable user-friendly platform to develop complete and interactive decision support to select the best area for the growth of stone fruit trees. The platform improves the technician’s decision-making, enhances crop productivity, and allows the choice of varieties according to the most optimal climatic characteristics of the area, promoting sustainable agricultural production.

Due to the changing climatic conditions, this tool personalizes and makes it possible to adapt to these changes. The system does not solve any specific problem. However, it provides information to the technical manager about the climatic suitability to place the crops in the area with the best conditions, which will make the plant more adapted to the area and require fewer cultivation techniques (human influence) and thus achieve a more sustainable crop. The crop’s suitability determines productivity levels (kg per tree or hectare), not economic yield. The yield depends on each producer’s commercial channels and on the risks they want to assume when introducing varieties in climatically unsuitable areas, either due to lack of cold or frost.

From a functional point of view, the system has two optimization options that answer two questions:

1. What agroclimatic characteristics occur at a specific location? Thanks to the verification of agroclimatic, biophysical, phenological, or agronomic trends, it allows us to determine the agroclimatic evolution necessary to monitor a given varietal group.
2. Is it advisable to produce a specific varietal group in this area? A list of suitable varieties is provided for a certain period based on each varietal group’s necessary cold requirements; furthermore, the probability of frosts that can occur in the area are estimated.

In the future, thanks to the large volume of data generated, it is possible to add Deep Learning techniques to predict the daily behavior of other variables such as reference evapotranspiration or to create other risk models for extreme events. We also plan to apply remote sensing techniques to map the physiological status of varietal groups globally and at local resolution.

## Figures and Tables

**Table 1:** Automatic stations by municipality and total number of collected records (data collected from January 2000 to March 2021).

Province	Stations	Total Hourly Records
Albacete	3	513,497
Alicante	15	2,513,738
Almería	10	1,714,600
Badajoz	16	2,519,111
Cáceres	16	2,521,390
Castellón	9	1,429,819
Granada	10	1,592,717
Huelva	10	1,665,803
Huesca	19	2,665,967
Lérida	20	2,114,226
Logroño	12	1,526,732
Murcia	40	7,270,082
Navarra	18	2,481,964
Sevilla	19	3,095,196
Valencia	23	3,775,681
Zaragoza	21	2,840,312

**Table 2:** Quantification of chilling unit requirements for varietal groups of different stone fruit trees.

Crop	Acumulated Cold Base	Varietal Group	Heat Start
ine Peach tree	250	Very Low (<250)	125
Peach tree	500	Low (250–500)	250
Peach tree	750	Medium (500–750)	375
Peach tree	1000	Medium-High (750–1000)	500
Peach tree	1250	High (1000–1250)	625
Apricot tree	600	Low (500–700)	300
Apricot tree	800	Medium (700–900)	400
Apricot tree	1000	Medium-High (900–1100)	500
Apricot tree	1250	High (>1100)	600
Japanese plum	625	Low (500–700)	315
Japanese plum	800	Medium (700–900)	400
Japanese plum	1000	High (>1000)	500
Sweet cherry	625	Low (500–750)	315
Sweet cherry	825	Medium (750–1000)	415
Sweet cherry	1000	High (>1000)	500
ine			

**Table 3:** Stations in the Region of Murcia by climatic zone and their most representative average climatic data.

Climate Zone	Total Stations	Average Temperature	Average Accumulated Precipitation
Zone B	6	9.12 ${}^{\circ }$C	185 mm
Zone C	7	11.21 ${}^{\circ }$C	165 mm
Zone D	24	13.02 ${}^{\circ }$C	170 mm
Zone E	3	14.59 ${}^{\circ }$C	155 mm

## Data Availability

Data sharing not applicable.
